# Revolutionising acute aortic syndrome diagnosis: The role of artificial intelligence in non‐contrast computed tomography

**DOI:** 10.1002/ctm2.70630

**Published:** 2026-02-22

**Authors:** Qiqi Wang, Yujian Hu, Yan‐Jie Zhou, Mengyun Yao, Yangyan He, Yilang Xiang, Minfeng Xu, Hongkun Zhang

**Affiliations:** ^1^ Zhejiang University School of Medicine Hangzhou China; ^2^ Department of Vascular Surgery The First Affiliated Hospital of Zhejiang University School of Medicine Hangzhou China; ^3^ DAMO Academy, Alibaba Group Hangzhou China; ^4^ Hupan Laboratory Hangzhou China

1

Rapid and accurate diagnosis of acute aortic syndrome (AAS) is crucial.^1^ Without treatment, 40%–50% of patients die within 48 hours, and mortality increases by 1%–2% per hour of delay.^2^, ^3^ Therefore, any delay in diagnosis substantially worsens the prognosis.^1^ However, AAS is difficult to diagnose because its symptoms are nonspecific^4^ and routine tests are unreliable.^5^ Although aortic computed tomography angiography (CTA) is considered the gold standard for diagnosing AAS,^1^ it is expensive, carries risks such as anaphylaxis and nephrotoxicity from contrast agents,^5^ and is often not available in resource‐limited settings like China^6^ and other low‐ and middle‐income countries.^7^ Correct diagnosis of AAS currently relies heavily on clinical suspicion, as there is no simple, dedicated diagnostic algorithm.^1^ Noncontrast CT is a convenient, cost‐effective, and widely used imaging modality, and its potential for screening AAS has been explored previously.^8^ However, it still lacks adequate sensitivity and specificity for accurate AAS diagnosis.^9^ In this context, artificial intelligence (AI) applied to non‐contrast CT scans offers a promising approach.

Here, we developed an AI‐based warning system called iAorta.^10^ The system is based on a deep learning model trained on 3350 aortic CTA scans. It uses non‐contrast phase images supervised by diagnostic labels and lesion segmentations transferred from arterial phase scans via image registration. The model detects acute aortic syndrome at the patient level, segments the aorta and true lumen precisely, and locates lesion regions with high reliability. To improve interpretability, the system produces activation maps that visually highlight areas corresponding to pathological regions, providing slice‐level visual explanations for different disease subtypes (Figure 1a). Integrated into a browser‐server platform, iAorta processes data in real time, sends alerts to radiologists, and supports clinical decision‐making, improving both speed and accuracy of AAS detection in emergency settings.

**FIGURE 1:**
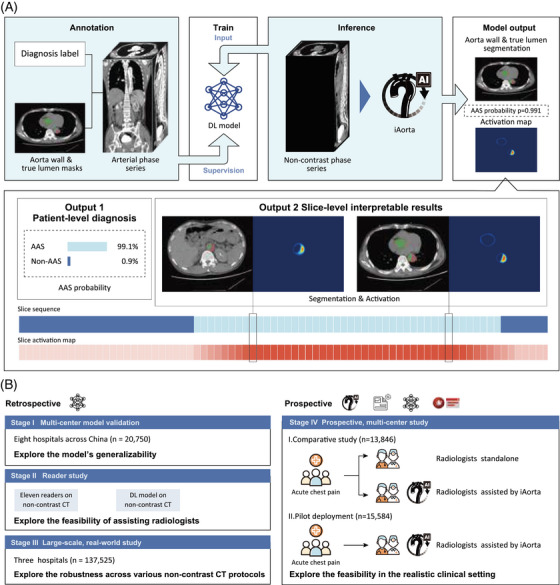
**Overall study design and model pipeline**. (a) Schematic of the model pipeline. The model is trained using supervision from arterial phase series, which are annotated with patient‐level diagnoses and segmentation masks for the aortic wall and true lumen. During the inference stage, the model processes non‐contrast computed tomography (CT) series to output the probability of acute aortic syndrome (AAS), alongside segmentation masks and activation maps that localise suspected lesion areas. (b) Overview of the multi‐stage evaluation pipeline. The retrospective phase focuses on generalizability across centres (Stage I, *n* = 20,750), the feasibility of assisting radiologists (Stage II), and robustness in a large‐scale real‐world setting (Stage III, *n* = 137,525). The prospective phase (Stage IV) investigates the iAorta system—integrating phase selection, the AI model, and an alert module—within clinical workflows, using a comparative analysis (*n* = 13,846) and pilot deployment (*n* = 15,584) to assess its efficacy in daily clinical practice.

The performance of iAorta has been rigorously validated (Figure 1b). In a multicenter model validation across eight centres (*n* = 20,750), the model achieved a sensitivity of 0.954–0.984 and specificity of 0.929–0.947 for detecting AAS on non‐contrast CT. In a large‐scale real‐world retrospective study (*n* = 137,525), the model showed consistent performance across different scanning protocols, with sensitivity of 0.913–0.942 and specificity of 0.991–0.993. In a reader study, the diagnostic sensitivity of medical trainees increased by 52.4% (from 0.603 to 0.919) with the model's assistance, reaching a level similar to that of specialist experts.

During a pilot implementation at a Chinese hospital, iAorta was integrated into the routine workflow, automatically analysing CT images from the hospital PACS. It identified patients at risk of AAS and provided real‐time alerts to radiologists via an interactive interface. Between 20 December 2024, and 20 February 2025, iAorta helped detect 21 out of 22 AAS cases among 15,584 consecutive emergency department patients who underwent non‐contrast CT, missing only one penetrating atherosclerotic ulcer. The system demonstrated high diagnostic accuracy, with sensitivity of 0.955 (95% confidence interval [CI] 0.864–1.000) and specificity of 0.994 (95% CI 0.993–0.995). The average time to diagnosis for confirmed AAS cases was reduced to 102.1 min (range 75–133), while a study based on the International Registry of Acute Aortic Dissection reported the median time from arrival at the emergency department to diagnosis for Stanford type A aortic dissection, even with typical symptoms, is 4.3 h.^11^ Thus, iAorta contributes to a substantial reduction in diagnostic delay. (See Figure 1b).

While the potential of using non‐contrast CT for screening AAS has been explored previously,^8^ the diagnostic performance of non‐contrast CT alone has not been well characterised. iAorta changes the screening approach for AAS by making non‐contrast CT a primary, efficient, and reliable clinical decision tool rather than only an auxiliary imaging method. For many patients with atypical chest pain, iAorta allows clinicians to make triage decisions using the most readily available initial imaging information. It helps direct emergency CTA resources to high‐risk patients while providing low‐risk patients with a reliable exclusion based on its high negative predictive value, reducing unnecessary invasive procedures and related risks (Figure 2a).

**FIGURE 2:**
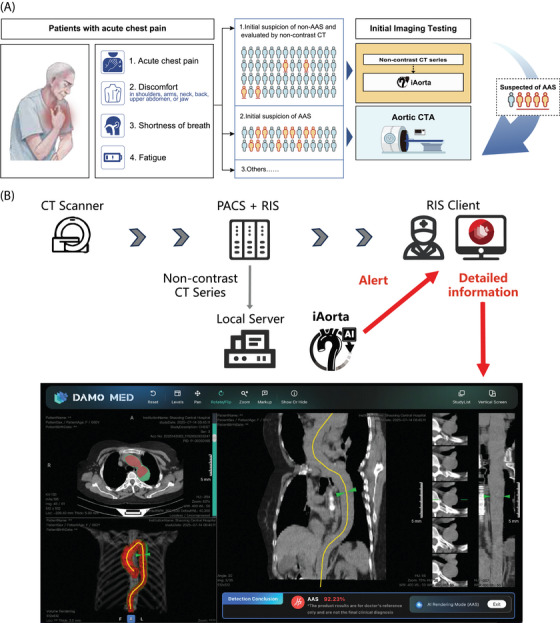
**Clinical application and workflow integration of iAorta**. (a) Clinical application scenario. The diagnosis of acute aortic syndrome (AAS) poses a notable challenge within the ED owing to its nonspecific clinical symptoms. In China, more than half of the patients with acute chest pain are initially suspected of less critical illnesses and thus received non‐contrast computed tomography (CT) scans as the initial imaging test. iAorta can rapidly and accurately identify patients with suspected AAS from this population of individuals undergoing non‐contrast CT scans, which can assist radiologists and physicians in making informed clinical decisions. (b) Flowchart describing the process of the seamless integration of iAorta into the existing clinical workflow. We collect data from Picture Archiving and Communication System (PACS) according to Radiology Information System (RIS) records, and the iAorta system is run on the local private server. iAorta system pushes alerts and results to the RIS Client. Radiologists could receive pop‐up alerts and review detailed results in the interactive interface, including abnormality probabilities, aorta wall and true lumen segmentation masks, and activation maps indicating possible lesion area.

iAorta also provides radiologists and emergency physicians with clear, verifiable information to support clinical judgment through visualisation outputs. This transparency allows the AI to act as a decision‐support tool whose reasoning can be understood and checked, rather than a “black box”. By supporting collaboration between clinical expertise and AI analysis, iAorta encourages long‐term integration into routine practice, addressing a key barrier to sustainable AI use in medicine^12^ (Figure 2b).

What's more, iAorta addresses the critical “last mile” of AI adoption by serving as a comprehensive equaliser across both healthcare infrastructure and clinical expertise.^13^ It ensures accessibility and scalability by using widely available non‐contrast CT equipment. It allows advanced diagnostic capabilities to extend from top‐tier academic centres to resource‐limited community hospitals, effectively mitigating regional disparities in healthcare access. By minimising inter‐observer variability^14^ and the reliance on individual experience, the system ensures consistent, expert‐level accuracy for high‐stakes acute conditions, optimising patient management.

Looking ahead, iAorta establishes a reproducible framework for unlocking the latent diagnostic value of non‐contrast CT. This model serves as a blueprint for screening other time‐sensitive conditions, such as non‐ST‐segment‐elevation acute coronary syndrome, pulmonary embolism and oesophageal rupture,^4^ paving the way for a comprehensive “one‐scan, multiple‐screening” ecosystem.^15^ Eventually, this evolution transforms routine imaging from a specialised diagnostic aid into a generalised critical care platform, maximising existing infrastructure to revolutionise emergency diagnostic efficiency.

## 3 CONFLICT OF INTEREST STATEMENT

The authors declare no conflict of interest.
